# Factors Altering Pyruvate Excretion in a Glycogen Storage Mutant of the Cyanobacterium, *Synechococcus* PCC7942

**DOI:** 10.3389/fmicb.2016.00475

**Published:** 2016-04-05

**Authors:** Phoebe J. Benson, Diane Purcell-Meyerink, Charles H. Hocart, Thy T. Truong, Gabriel O. James, Loraine Rourke, Michael A. Djordjevic, Susan I. Blackburn, G. D. Price

**Affiliations:** ^1^Research School of Biology, Plant Sciences, Australian National University, CanberraACT, Australia; ^2^North Australia Marine Research Alliance, Arafura Timor Research Facility, DarwinNT, Australia; ^3^Heliase Genomics, University of AucklandAuckland, New Zealand; ^4^CSIRO National Research Collections Australia, HobartTAS, Australia

**Keywords:** cyanobacteria, pyruvate excretion, overflow metabolism, photosynthesis, physiolology and metabolism, nitrogen deprivation, biotechnology of microorganisms

## Abstract

Interest in the production of carbon commodities from photosynthetically fixed CO_2_ has focused attention on cyanobacteria as a target for metabolic engineering and pathway investigation. We investigated the redirection of carbon flux in the model cyanobacterial species, *Synechococcus elongatus* PCC 7942, under nitrogen deprivation, for optimized production of the industrially desirable compound, pyruvate. Under nitrogen limited conditions, excess carbon is naturally stored as the multi-branched polysaccharide, glycogen, but a block in glycogen synthesis, via knockout mutation in the gene encoding ADP-glucose pyrophosphorylase (*glgC*), results in the accumulation of the organic acids, pyruvate and 2-oxoglutarate, as overflow excretions into the extracellular media. The Δ*glgC* strain, under 48 h of N-deprivation was shown to excrete pyruvate for the first time in this strain. Additionally, by increasing culture pH, to pH 10, it was possible to substantially elevate excretion of pyruvate, suggesting the involvement of an unknown substrate/proton symporter for export. The Δ*glgC* mutant was also engineered to express foreign transporters for glucose and sucrose, and then grown photomixotrophically with exogenous organic carbon supply, as added 5 mM glucose or sucrose during N- deprivation. Under these conditions we observed a fourfold increase in extracellular pyruvate excretion when glucose was added, and a smaller increase with added sucrose. Although the magnitude of pyruvate excretion did not correlate with the capacity of the Δ*glgC* strain for bicarbonate-dependent photosynthetic O_2_ evolution, or with light intensity, there was, however, a positive correlation observed between the density of the starter culture prior to N-deprivation and the final extracellular pyruvate concentration. The factors that contribute to enhancement of pyruvate excretion are discussed, as well as consideration of whether the source of carbon for pyruvate excretion might be derived from photosynthetic CO_2_ fixation or from remobilisation of existing carbon stores.

## Introduction

Cyanobacteria consume CO_2_ and convert it to carbohydrates, lipids, and proteins and have received much attention as a source of bioproducts and biofuels (Ducat et al., 2012). The inexpensive inputs required to operate cyanobacterial metabolic processes, combined with their capacity for genetic engineering, make them ideal candidates for investigating the potential bioproduction of valuable organic compounds.

The fundamental challenge of bioproduction from cyano bacteria lies in the ability to manipulate cellular partitioning of macromolecules to enhance the ratio of desired product-to-biomass. Enzymatic activities and the consequent carbon flux may be driven toward product synthesis by the application and manipulation of the environmental conditions of the culture system, such as nutrient availability (Gonzalez-Fernandez and Ballesteros, 2012). Nutrient limitation markedly decreases cell growth, which allows for photosynthetically fixed carbon to be transiently redirected to products of interest (Schwarz and Forchhammer, 2005).

Nitrogen is required for the synthesis of proteins, genetic material and other cell structures. If environmental nitrogen availability is too low to cover nitrogen demand, cyanobacteria redirect carbon to internal stores to maintain a basal growth rate and to partition excess carbon until nitrogen is re-applied (Gorl et al., 1998). This results in an up-regulation of sugar catabolic pathways and mass accumulation of carbon reserve polymers, chiefly glycogen, to as much as 40–60% of the dry weight of *Synechococcus elongatus* (Allen, 1988; Schwarz and Forchhammer, 2005; Hickman et al., 2013). Glycogen is synthesized by the sequential actions of ADP-glucose pyrophosphorylase (AGPase), glycogen synthase and branching enzyme (amylo (α1 → 4) to (α1 → 6) transglycosylase). ADPase (encoded by *glgC*) catalyses the synthesis of ADP-glucose from glucose-1-phosphate and ATP. This represents a major rate-controlling step of glycogen synthesis (Preiss, 1984). Mutants deficient in *glgC* (Δ*glgC*) have been constructed and characterized under both nitrogen sufficient and depleted conditions (Suzuki et al., 2010; Carrieri et al., 2012; Grundel et al., 2012; Hickman et al., 2013; Jackson et al., 2015).

When nitrogen deprived, the cyanobacterium *Synechocystis* sp. PCC 6803 Δ*glgC* was found to accumulate the organic acids pyruvate (0.4–0.6 mM, 35–52 mg/L after 48 h) and 2-oxoglutarate (0.14–0.17 mM, 20–24 mg/L after 48 h), which accumulate in the extracellular medium (Carrieri, et al., 2012; Grundel, et al., 2012). This elevation of pyruvate and 2-oxoglutarate has been observed previously, in cultures of *Streptomyces*, a genus of Gram positive bacteria, grown on glucose (Madden et al., 1996) and in *Alcaligenes eutrophus*, a proteobacterium, when impaired in polyhydroxybutyrate (PHB) synthesis and grown in nutrient-limited medium with excess carbon (Steinbuchel and Schlegel, 1989). However, until now, N-deprivation in *Synechococcus* PCC7942 Δ*glgC* has not been observed to result in pyruvate production.

Stored lipid is another potential over-flow destination when considering a Δ*glgC* mutant subject to N-deprivation. Recent investigations with a Δ*glgC* strain of *S. elongatus*-under N-deprivation reported a minor increase in the major fatty acids of *S. elongatus* membrane lipids, palmitic acid, palmitoleic acid, and stearic acid, after 4 h of nitrogen depletion. This could indicate a limited redirection of flux into the lipid biosynthesis pathway. Typically, the nutrient-replete lipid content of cyanobacteria (5–13% of dry cell weight, DCW, with an average of 8% DCW) (Griffiths and Harrison, 2009) is too low to be considered a viable biofuel source, especially when compared to lipid accumulation in green algae (13–31% DCW, with an average of 23% DCW) (Griffiths and Harrison, 2009). However, with the removal of alternative carbon storage pathways, lipid biosynthesis could be favored over longer time frames, and the glycogen deficient strain thus represents a viable basis for investigation.

In theory, limitations imposed on the photosynthetic rate of carbon fixation and carbon re-allocation can be investigated by oversupplying exogenous carbon compounds and growing cultures photomixotrophically. *S. elongatus* is an obligate phototroph lacking the ability to take up and consume fixed carbon compounds to increase biomass, however, it was successfully engineered (McEwen et al., 2013) for continuous growth under diurnal conditions by engineering sugar transport systems into this cyanobacterium. Of the engineered sugar transport systems, the most productive were: *galP*, a MFS-type D-galatactose/H^+^ transporter from *Escherichia coli*, grown on glucose; and *cscB*, a MFS-type D-sucrose/H^+^ transporter in conjunction with *cscK*, a fructokinase from *E. coli*, grown on sucrose (McEwen et al., 2013).

In this present study, the redirection of carbon flux in the AGPase deficient mutant (*ΔglgC*) of the cyanobacterial species *S. elongatus* was investigated. It was hypothesized that, similar to *Synechocystis* 6803 Δ*glgC*, there would be an overflow of pyruvate and 2-oxoglutarate into the external medium. The excretion of 2-oxoglutarate and the TCA cycle intermediates, succinate, and fumarate, has previously been reported for *S. elongatus*, though curiously, the presence of pyruvate in the extracellular media was not detected (Hickman et al., 2013). As the metabolic pathways of *S. elongatus ΔglgC* and *Synechocystis* 6803 Δ*glgC* should be very similar, it would be reasonable to expect pyruvate to also be secreted. Indeed, pyruvate excretion was observed in our *ΔglgC* mutant, and it was found to be effected by alteration of the culture medium pH and the re-addition of minimal amounts of reduced nitrogen across a nitrogen deprived time course. Another aspect was related to the possible internal source of these carbon skeletons for pyruvate production. Previous studies using ^13^C-labeled bicarbonate and NMR spectroscopy (Carrieri et al., 2012) reported that the organic acids present in the extracellular medium of *Synechocystis* Δ*glgC* were synthesized *de novo* and not from the remobilisation of previously stored cell material. It is predicted that the supply of reduced carbon is the limiting factor for pyruvate synthesis. Thus, if pyruvate is derived from photosynthetically fixed carbon, there should be a correlation between the rates of photosynthesis and pyruvate excretion. To test these hypotheses, the net rate of photosynthetic oxygen evolution was compared to the supernatant concentration of pyruvate when cells were subjected to nitrogen deprivation. In addition, *S. elongatus*-Δ*glgC* was engineered to express glucose (*galP* from *E. coli*) and sucrose (*cscKB* from *E. coli*) transporters and was grown photomixotrophically with glucose or sucrose to assess the capacity for elevated pyruvate production. Combined, our data suggests, for *S. elongatus*-Δ*glgC*, that excreted pyruvate is not derived from new photosynthetic carbon fixation. Rather the pyruvate appears to be synthesized from, and limited by, the availability of previously stored carbon.

## Materials and Methods

### Strain Development

Gene deficient strains were developed by replacing the WT gene with the appropriate antibiotic resistance gene using homologous DNA cross-over (Elhai and Wolk, 1988). For deletion inactivation of *glgC* in *Synechococcus* PCC7942 (Synpcc7942_0603; img.jgi.doe.gov), designated Δ*glgC*, the Tn903 kanamycin resistance (Tn903) marker was inserted into the PstI site 724 bp downstream of the start codon of *glgC*, using a left flank of 714 bp and a right flank of 973 bp downstream of PstI (Rae et al., 2011). The *sps* gene (Synpcc7942_0808; img.jgi.doe.gov) in *Synechococcus* PCC7942 was inactivated by replacing bases 496–1677 with the spectinomycin resistance marker (Elhai and Wolk, 1988) using a left flank of 1–496 bp of *sps* and the right flank of 1677–2172 bp of *sps*. *Synechococcus* PCC7942 cells typically maintain 5–15 copies of the genome (Jain et al., 2012) so putative mutants were given time to segregate under drug selection and checked by PCR to ensure all copies of the chromosomes were homogenous (Klughammer et al., 1999). Stock cell lines were maintained on BG11 plates, solidified using 1.0% agar, with the appropriate antibiotic. Plates were incubated at 30°C, 3% CO_2_ and under constant illumination with cool white fluorescent light of 40–50 μmol photons m^-2^ sec^-1^ (Badger and Price, 1989). To construct the sugar transporter strains, gene sequences for *galP* (glucose) and *cscKB* (sucrose) were derived from *E. coli* essentially as previously detailed (McEwen et al., 2013). Restriction enzyme sites for EcoRI, NcoI, XbaI, and HindIII were added to the ends of the sequence *in silico* and restriction sites for these enzymes were removed if present within the gene sequence. The sequences were then commercially synthesized and delivered in the pUC57 plasmid vector. The transporter genes were cloned into the NcoI and XbaI restriction sites in the shuttle vector pSE2 downstream of a *lac* promoter (Maeda et al., 1998). pSE2-transporter plasmids were transformed into *S. elongatus*-Δ*glgC* and grown under antibiotic selection (spectinomycin at 10 μg ml^-1^). To initiate transporter expression, 0.1 mM of isopropyl β-D-1-thiogalactopyranoside (IPTG) was added to the liquid starter cultures 2 h prior to the initiation of experimental conditions. Cultures were prepared as below, with the addition of 5 g/L of the appropriate saccharide and re-addition of 0.1 mM IPTG to the culture media at the commencement of the time course.

### Growth Conditions in Liquid Media

Liquid cultures of all *S. elongatus* strains were grown at 30°C, under constant illumination in liquid BG11 medium with 20 mM HEPES buffered to pH 8 with KOH, unless otherwise stated. Cultures, in 120 mL test tubes, were continuously aerated with 3% CO_2_ and illuminated with cool white fluorescent light of 70–75 μmoles photons m^-2^ s^-1^. Chlorophyll content was determined in methanol (Porra et al., 1989). To prepare cultures for experimental conditions, cells were grown in 100 mL of standard BG11 to an OD_730_ of 1.5–2.0. To normalize cultures, 18–24 h before the commencement of experimental conditions, cultures of the same strain were combined and diluted to approximately 0.8-1.2 OD_730_ and redistributed into sterile growth tubes. Standard experimental conditions for induced N-deprivation (BG11_0_) were established by centrifugation (10 min at 4000 *g*; RT) and resuspension in 50 mL of sterile BG11/BG11_0_ media to an OD_730_ of 1.0 ± 0.15. For high pH treatments under N-deprivation, log phase cells were centrifuged and resuspended to an OD_730_ of 1.0 ± 0.2 in 50 mL BG11_0_ media buffered with 20 mM CHES-KOH to pH10.

### Culture Assessment

**FIGURE 1:**
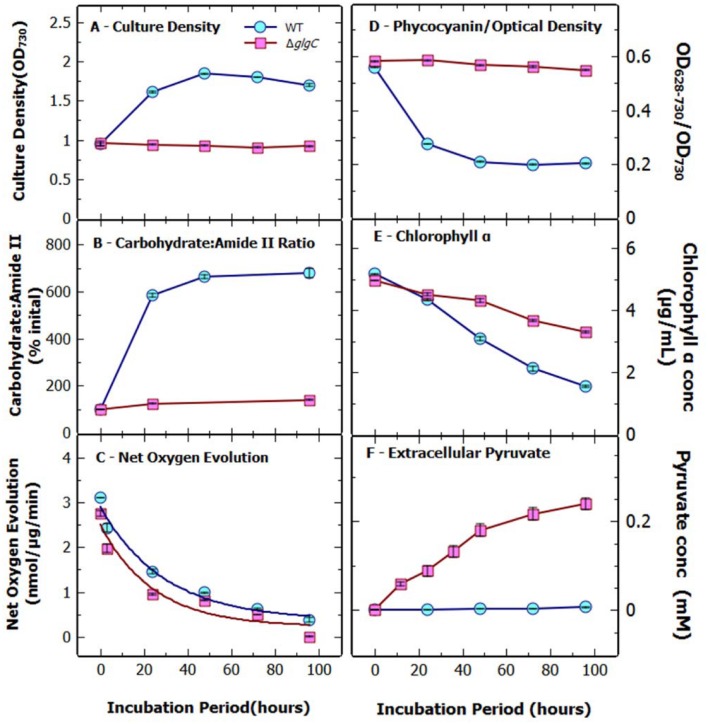
**Physiological assessment of *Synechococcus elongatus* WT and *ΔglgC* cultures under N-starvation.** The **(A)** culture density (A_730_), **(B)** carbohydrate : amide II ratio, **(C)** net oxygen evolution, **(D)** ratio of phycocyanin to optical density, **(E)** chlorophyll *a* concentration, and **(F)** extracellular pyruvate concentration of *S. elongatus* WT and *ΔglgC* cultures over 96 h nitrogen starvation. Data indicate mean ± SE (*n* = 4) for four biological replicates.

Growth of cells was determined by measuring the optical density of cell suspensions at 730 nm (OD_730_). Similar to previous studies (Suzuki et al., 2010), a cell suspension with an OD_730_ of 1.0 contained 1.1 (±0.1) × 10^8^ cells ml^-1^ (average ± SD), with no discernible difference between the WT and mutant cells when grown under N-replete conditions. Thus, when cells were setup at a standard density of OD_730_ of 1.0, for N-free conditions, that cells per mL and chl per mL were near identical (**Figure 1**). The quantity of phycocyanin in the culture was assessed by determining the ratio of absorbance at 628 nm, the phycocyanin absorbance peak (Grundel et al., 2012), to the cell density (OD_730_), of the cell suspension. Fourier transform infrared (FTIR) spectroscopy was used to assess changes in protein and carbohydrate profiles in cells, as previously described (Stehfest et al., 2005). At time zero, log phase cells in nutrient sufficient BG11 were centrifuged and resuspended to an OD_730_ of 1.0 ± 0.1 in 50 mL BG11 – NO_3_ media buffered with 20 mM HEPES to pH8. Cultures were aerated with 3% CO_2_ at 30 °C, under continuous illumination of 70–75 μmoles photons m^-2^ s^-1^. Cultures were sampled at 0, 24 and 48 h following N-deprivation and stored at -20°C. For FTIR analysis, cells were washed and resuspended in H_2_O to an OD_730_ of approximately 10. A 50 μL aliquot was placed in each well of a 96 well plate and dried at 60°C for 2 h. Spectra were collected using an FTIR spectrometer (VERTEX 80, Bruker Optics, Karsruhe, Germany) coupled with a microplate extension (HTS-XT, Bruker Optics, Karsruhe, Germany) and fitted with a KBr beam splitter. The absorbance spectra were acquired over 64 scans between 900 and 4000 cm^-1^ at a spectral resolution of 4 cm^-1^. The pinhole aperture on the sensor was set at a diameter of 8 mm. To minimize differences in spectra due to baseline shifts, the spectra were baseline corrected using the “rubber band” algorithm, excluding CO_2_ peaks, of the OPUS control software (version 7.2, Bruker Optik Gmbtl). The OPUS control software package was then used to measure the absorbance of the following functional groups: C = O in lipids (1745 cm^-1^); amide C = O (1655 cm^-1^, amide I – protein); amide N-H (1540 cm^-1^, amide II) and C-O-C in carbohydrates (1200–900 cm^-1^) (Stehfest et al., 2005).

### Measurement of Net Photosynthetic O_2_ Evolution

For assessment of photosynthetic responses to external Ci concentration, photosynthetic oxygen evolution was measured using membrane-inlet mass spectrometry (Sultemeyer et al., 1995; Woodger et al., 2003). Unless stated otherwise, measurements were conducted at 30°C at a saturating light intensity of approximately 800 μmoles photons m^-2^ s^-1^. Cells were collected by centrifugation, then assayed in medium was identical to the growth medium with the exception that 20 mM HEPES–KOH was replaced with 50 mM Bis–Tris propane, pH 7.9, and 19 mM NaNO_3_ was replaced with 20 mM NaCl. In some cases when the media contained no nitrogen, maximal photosynthetic rates were determined in cells cultures transferred directly to the cuvette. Cultures were assayed at a chlorophyll *a* density of 2 μg/mL.

### Organic Acid Measurement

Cultures were examined for contamination by plating 50 μl of a 1/100 dilution from the final time point of every sample on Luria Bertani (LB) plates. So as not to underestimate the metabolite levels in the media, cultures were excluded from further analysis if the number of contaminates present was >50, representing a contamination level of >0.01% in 1 OD_730_ cultures. Culture supernatant was collected for the pyruvate assay, by centrifugation, and stored at 4°C. Supernatant pyruvate concentration was measured using an adapted fluorometric biochemical assay (Zhu et al., 2010). An α-ketoglutarate assay kit (Sigma–Aldrich, MAK054) was used to measure 2-oxoglutarate concentration. Assays were completed on black Nunc-Immuni MicroWell 96-Well polystyrene plates (Sigma–Aldrich, P98741). Fluorescence was measured at Ex/Em = 540/590 nm using FLUOstar Optima (BMG Labtech, software version 1.32 R2). The protein content of the supernatant was assessed using the Coomassie (Bradford) Protein Assay Kit (Thermo Scientific, USA). For pyruvate stability studies, exogenous sodium pyruvate was added to BG11_0_ media or water to a final concentration of 10 mM, then treatment aliquots were taken and stored in 1.5 mL Eppendorf tubes, at the various temperatures and freezing conditions. Snap-freezing was achieved by submersion of samples in liquid nitrogen. After 24 h samples were bought to room temperature and the pyruvate concentration was measured. The stability of exogenously added pyruvate in culture supernatant over 18 h in different growth conditions with *S. elongatus* 7942 WT cells was examined. At time zero, log phase cells in nutrient sufficient BG11 were centrifuged and resuspended to an OD_730_ of 1.0 ± 0.1 in 100 mL BG11/BG11_0_ media buffered with 20 mM HEPES to pH 8 and containing 10 mM sodium pyruvate. Cultures were aerated with 3% CO_2_ at 30°C. Light intensity, where applicable, was 70–75 μmoles photons m^-2^ s^-1^. The pyruvate concentration in the supernatant was measured within 1 h of collection.

### Lipid Analysis

Cultures were sampled in triplicate at 0, 24, 48, and 72 h (70–100 mL, OD of 0.8–1.2). Cell pellets were harvested by centrifugation (3220 *g*, 20 min at 25°C), frozen in liquid nitrogen and then dried using a vacuum centrifuge over night or until dry. Dry weight was determined by weighing tared vials. Fatty acid methyl esters (FAMEs) were prepared by a modification of the direct trans-esterification method as described by Lewis et al. (2000). The dried sample (2–10 mg) was weighed into 10 mL screw-top Teflon tubes (Nalgene) and the pellets broken up using a metal spatula. Methanolic hydrochloric acid (1 mL, 3N, Sigma–Aldrich), chloroform (100 μL) and the internal standard (60 μL, 12.56 mg.mL^-1^ heptadecanoic acid, Sigma) were added (Christie, 2003). The cells were mixed with the reaction solution and then heated at 90°C for 60 min. The solution was set aside to cool, then 2 mL of water was added to each tube and the FAMEs extracted using hexane:chloroform (4:1 v/v, 3 × 2 mL). The FAME extract was concentrated under nitrogen and transferred to GC/MS auto-sampler vials for analysis. The position of double bonds in the fatty acids were determined from the mass spectra of the 4,4-dimethyloxazoline (DMOX) derivatives. The preparation of these derivatives and the gas chromatography–mass spectrometry (GC/MS) analysis and data processing was carried out as described previously (James et al., 2011).

## Results

### Phenotypic Response of *S. Elongatus* Δ*glgc* under a Switch to N-deprivation

Cells of WT and the Δ*glgC* strain were setup at a standard density of OD_730_ of 1.0, at the onset of N-free conditions, ensuring that that cells per mL and chlorophyll per mL were near identical (**Figure 1**). This also ensured that levels of extracellular excretion of pyruvate were assessed at a standard volume of growth media irrespective of later changes to cell OD per cell and chlorophyll content. Under conditions of a switch to N-deprivation, *S. elongatus* Δ*glgC* showed a cessation of growth, a lack of cell bleaching, a decline in oxygen evolution rates and lack of carbohydrate accumulation over a 96 h period (**Figure 1**). By comparison, WT *S. elongatus* cells completed a doubling of OD before cessation of growth (**Figure 1A**). This type of growth cessation and non-bleaching of the *glgC* mutants under N-deprivation has been seen previously for *Synechocystis* sp. PCC6803 (Carrieri et al., 2012) and *S. elongatus* (Hickman et al., 2013). Fourier transfer infrared spectroscopy (FTIR) was used to monitor the cellular accumulation of carbohydrate and lipid (Stehfest et al., 2005), and showed an almost sixfold increase in the carbohydrate to amide ratio in the WT strain within 24 h of nitrogen starvation (**Figure 1B**). This compared to an increase of only 1.2-fold for the Δ*glgC* strain. The FTIR spectrum for Δ*glgC* displayed minimal alteration in the internal carbohydrate profile over 96 h of N-deprivation (**Supplementary Figure S1**). Photosynthetic capacity, measured as net oxygen evolution rates, indicated that Δ*glgC* cells had a significantly lower photosynthetic capacity across the time course compared to WT (**Figure 1C**). However, the rate of decrease, when expressed as a fraction of the non-nitrogen deprived net oxygen evolution rate, was comparable between the two strains, reaching approximately 30% of the initial rate after 48 h (results not shown).

Cell beaching was visible in WT cultures within 24 h of N-deprivation, whilst there was no visible bleaching of the Δ*glgC* cells over 96 h. This was confirmed by the rapid decline in the ratio of the absorbance of phycocyanin to optical density in the WT, which was not seen in the mutant (**Figure 1D**). The chlorophyll *a* concentration decreased in both strains, however, the loss was more rapid in the WT, decreasing to approximately 1.5 μg/mL after 96-h, in comparison to the mutant, which was 3.2 μg/mL after 96-h (**Figure 1E**).

### Pyruvate Is Excreted by the *S. elongatus ΔglgC* Mutant under N-deprivation

In contrast to previous results (Hickman et al., 2013) we found that a *ΔglgC* strain is capable of high levels of pyruvate excretion under N-deprivation (**Figure 1F**). Pyruvate stability was investigated by dissolving a known concentration (10 mM) of sodium pyruvate in BG11 medium (**Supplementary Figures S2A–C**). We first established that under standard growth conditions (3% CO_2_, 30°C, light intensity 70–75 μmoles photons m^-2^ s^-1^), with WT *S. elongatus* cells, that added pyruvate was stable and that cells could not consume pyruvate under N-deprivation or when incubated in darkness. However, when the supernatant samples were frozen and thawed, a large and variable decrease in the measured pyruvate concentration was observed. Snap-freezing in liquid nitrogen amplified this response and the decrease was greater in BG11 media than in distilled water. The storage of supernatant samples at 4°C in a refrigerator had no effect on pyruvate concentration over 96 h. Similar experiments were conducted to investigate 2-oxoglutarate stability and it was found to be stable across all growth and storage conditions, however, 2-oxoglutarate also displayed minor variability in concentration when snap-frozen in liquid nitrogen (results not shown).

The presence of pyruvate in the supernatant of N-deprived *S. elongatus* Δ*glgC* cell cultures was readily detected using a coupled enzyme biochemical assay (Zhu et al., 2010) (**Figure 1F**). Most pyruvate excretion occurred within the first 48 h of N-deprivation, reaching up to 0.25 mM at 48 h (**Figure 1F**); concurrently, the net rate of oxygen evolution decreased by 70% (**Figure 1C**). Excretion of detectable levels of pyruvate was not observed in the WT under N-deprivation or in the Δ*glgC*-mutant under nutrient sufficient conditions; the mutant grew at the same rate as WT under N-sufficiency (results not shown). 2-Oxoglutarate excretion followed the same trend as for pyruvate (**Supplementary Figure S3**). No protein was detected in the supernatant of the N-deprived cultures over 4 days (results not shown) indicating that the cells remained intact during N-deprivation.

Measurements of lipid content (FAME data) over 3 days of N-deprivation, in the three strains (WT, Δ*glgC*, *and* Δ*glgC* + sps) showed only minor differences, but no evidence of significant accumulation of lipid (**Supplementary Table S1**). This finding is consistent with recent results (Hickman et al., 2013), and indicates that although the required TCA cycle precursors, pyruvate and oxo-glutarate, are available in high abundance for potential lipid synthesis (**Figure 1F**, **Supplementary Figure S3**) none of this capacity results in fatty acid accumulation. This is consistent with lipid levels in cyanobacteria being surprisingly low at 5–13% of dry cell weight, under a range of conditions and a range of species (Griffiths and Harrison, 2009).

Further enhancement of pyruvate excretion by removing alternative carbon storage pathways was also briefly examined. It was suspected that direct metabolic flux, in the-Δ*glgC* strain, toward pyruvate production, it would be desirable to knockout pathways leading to other intracellular carbon stores. Glycogen synthesis and sucrose synthesis have very similar biosynthetic pathways and actively compete for substrates. A block in glycogen synthesis removes this competition and could lead to an increased flow of carbon toward sucrose accumulation. Thus, genetic manipulation to block sucrose synthesis pathways in *S. elongatus* Δ*glgC* may lead to an increase in pyruvate excretion under N-deprivation. Sucrose-phosphate-synthase, encoded by the *sps* gene, is the main sucrose synthesizing enzyme in cyanobacteria. A knockout of the *sps* gene abolishes sucrose accumulation (Hagemann and Marin, 1999). Since an additive block in the sucrose accumulation pathway could potentially heighten pyruvate excretion, a Δ*glgC/*Δ*sps* was also assessed. However, this double mutant was found to excrete less than 40% that of Δ*glgC* at 48 h (results not shown). This is likely a consequence of the detrimental effect the double mutant had on the growth and photosynthetic rates (results not shown). Further experiments with this double mutant were curtailed.

### Pyruvate Production Was Not Correlated to Photosynthetic Rate

**FIGURE 2:**
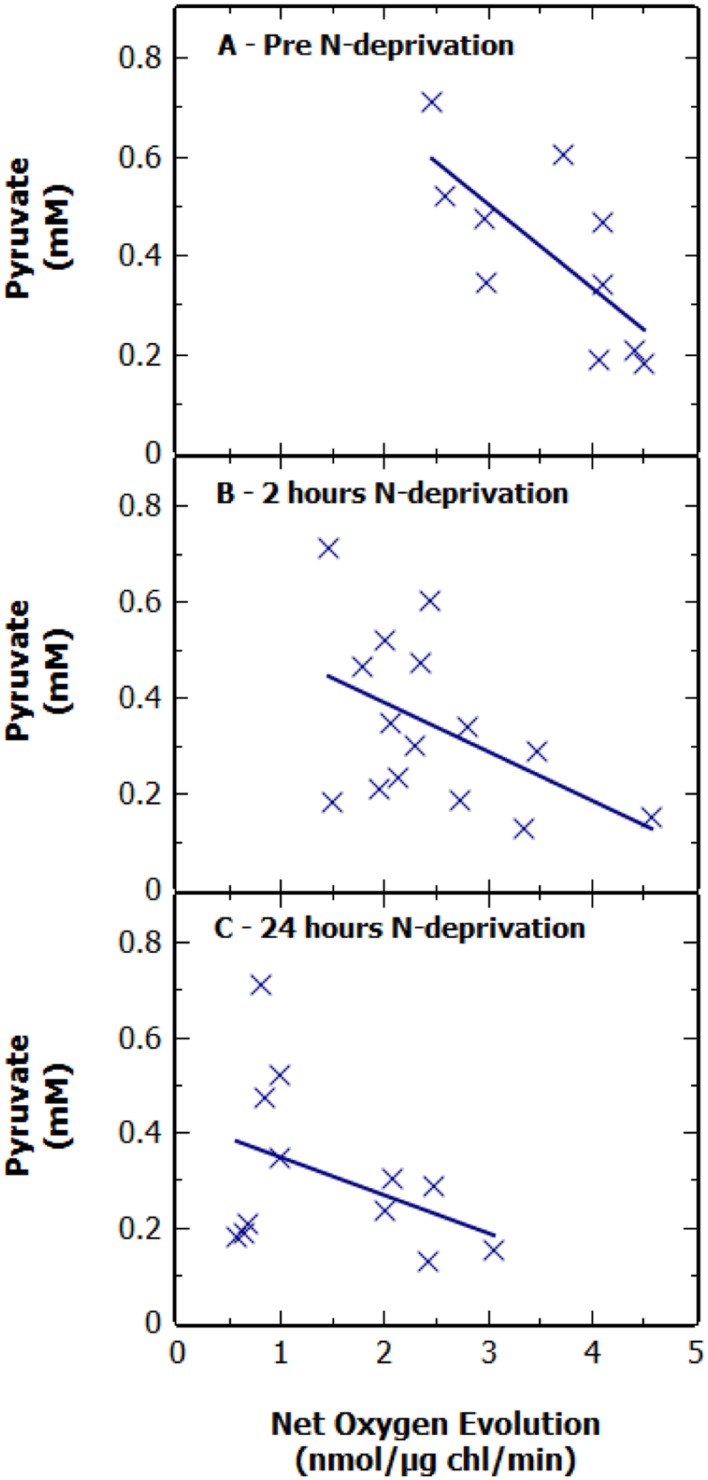
**Extracellular pyruvate concentration in cultures of *S. elongatus* Δ*glgC* after 48 h N-deprivation against net oxygen evolution.** Pyruvate levels are shown **(A)** pre-N deprivation, **(B)** 2 h after N-deprivation, and **(C)** 24 h after N-deprivation. Results represent a combination of multiple experiments. Pyruvate concentration is normalized to 1 OD_730_ at 0 h with an OD_730_ range of 0.84–1.2. Each point is displayed as the average of technical duplicates from pooled sets of experiments.

**FIGURE 3:**
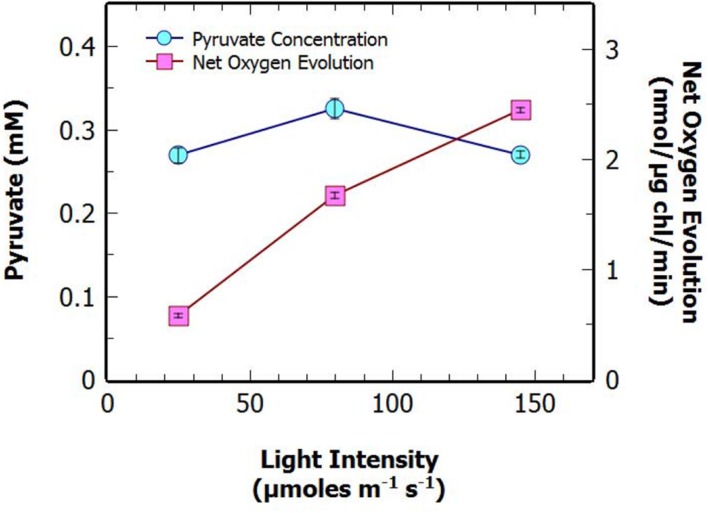
**Extracellular concentration of pyruvate, and net oxygen evolution at different light intensities for *S. elongatus* Δ*glgC* after 48 h in N-deprivation culture.** Data points represent mean ± SE (*n* = 3; the average of two technical duplicates for three biological replicates).

Previous studies have suggested that there may be a positive correlation between the photosynthetic rate of O_2_ evolution (as a surrogate for CO_2_ fixation) and rate of excretion of pyruvate (Carrieri et al., 2012; Jackson et al., 2015). To investigate this we measured the maximal bicarbonate-dependent, photosynthetic oxygen evolution rate, used here as a proxy for photosynthetic carbon fixation capacity, at various time points throughout N-deprivation using MIMS (**Figures 2A–C**). There was no apparent correlation between the net oxygen evolution rate of the culture, at 2 and 24 h, and pyruvate excretion after 48 h of N-deprivation (**Figures 2B,C**). Additionally, there was a slight negative correlation between the oxygen evolution of the culture prior to N-deprivation and the supernatant pyruvate concentration at 48 h (**Figure 2A**). This suggests that there is no strong relationship between the potential photosynthetic carbon fixation capacity and the rate of pyruvate excretion. To investigate this further, the net oxygen evolution rate of a non-nitrogen starved culture was measured at different light intensities and compared to the pyruvate production after 48 h of N-deprivation at the same light intensity (**Figure 3**). As expected the maximum rate of net oxygen evolution increased with light intensity, however, the pyruvate concentration remained relatively stable. This result is not consistent with the hypothesis that the carbon source for pyruvate is coming from newly fixed carbon. Instead, our results suggest that the supply of stored carbon, possibly from long-term pools, could be the source, and therefore, the limiting factor for pyruvate production.

### Supply of Reduced Carbon Is a Limiting Factor for Pyruvate Production

**FIGURE 4:**
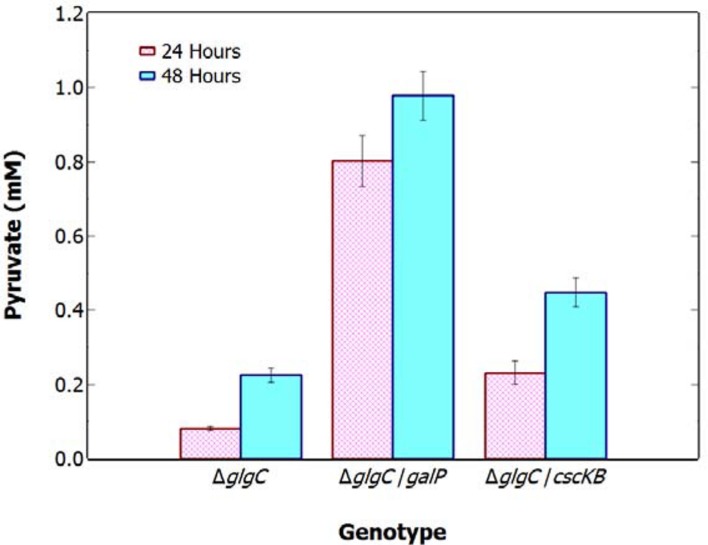
**Extracellular concentration of pyruvate in *S. elongatus* 7942 Δ*glgC*, Δ*glgC+galP*, and Δ*glgC+cscKB* after 48 h N-deprivation and photomixotrophic growth.** Data indicate mean ± SE (*n* = 3; the average of two technical duplicates for three biological replicates).

To investigate the limiting role of carbon supply on pyruvate production, Δ*glgC* was engineered to express saccharide transporters and grown photomixotrophically with excess carbon. The genes encoding the sugar transporters *galP* (glucose uptake) and *cscKB* (sucrose uptake) were inserted into the genome of the Δ*glgC* strain using the pSE2 shuttle vector under a *lac* inducible promoter. The greatest pyruvate excretion was seen in the Δ*glgC/galP* strain grown photomixotrophically under N-deprivation and added glucose, with a pyruvate concentration over fourfold greater than that of Δ*glgC* under N-deprivation, after 48-h (**Figure 4**). N-deprived Δ*glgC/cscKB* displayed a twofold increase in pyruvate production over Δ*glgC* when grown photomixotrophically. These results indicate that Δ*glgC* cultures have a higher capacity to produce pyruvate under N-deprivation if extra fixed carbon is available, demonstrating that pyruvate production is normally limited by the availability of endogenous reduced carbon.

### Pyruvate Production Is Positively Correlated to the Density of the Starting Culture

**FIGURE 5:**
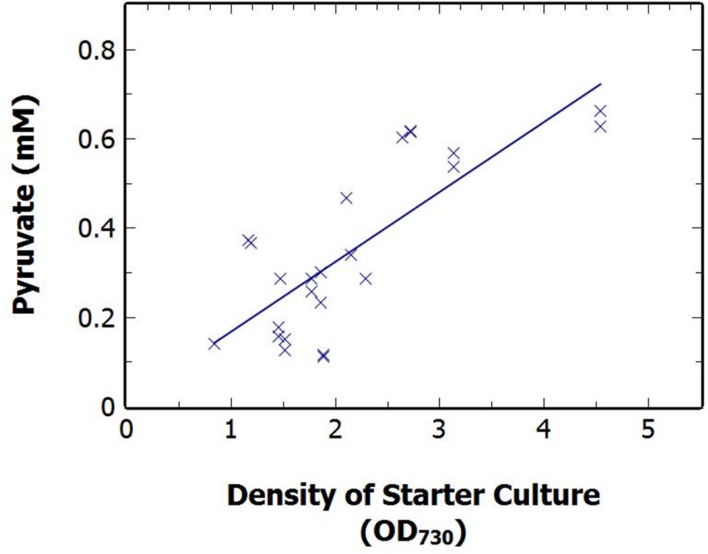
**The relationship between extracellular pyruvate at 48 h N-deprivation against the density of the starter culture density of *S. elongatus* Δ*glgC* just prior to dilution to a standard 1 OD_730_ at time zero.** Data points displayed as the average of technical duplicates from pooled sets of experiments.

A correlation existed between the density of the starting culture and the production of pyruvate over 48 h N-deprivation. *S. elongatus* Δ*glgC* cells were grown in nutrient sufficient BG11 media and diluted at a range of times, and to varying cell densities (OD_730_ 0.84–4.54). The cultures were harvested via centrifugation and resuspended in nitrogen-free BG11 media to produce 50 mL cultures of 1 ± 0.15 OD_730_. The extracellular concentration of pyruvate was measured after 48 h N-deprivation and found to be strongly correlated, with a Pearson R value of 0.79, to the OD_730_ of the starter culture (**Figure 5**). This strong positive correlation was also seen with the pyruvate production after 24 h (results not shown). The result suggests that older cultures, having greater reserves of stored carbon and stored protein, are best able to support pyruvate production from existing endogenous reserves, rather than from net fixed carbon due to photosynthesis.

### Pyruvate Production Was Increased by Alkalinity of the Culture Media

**FIGURE 6:**
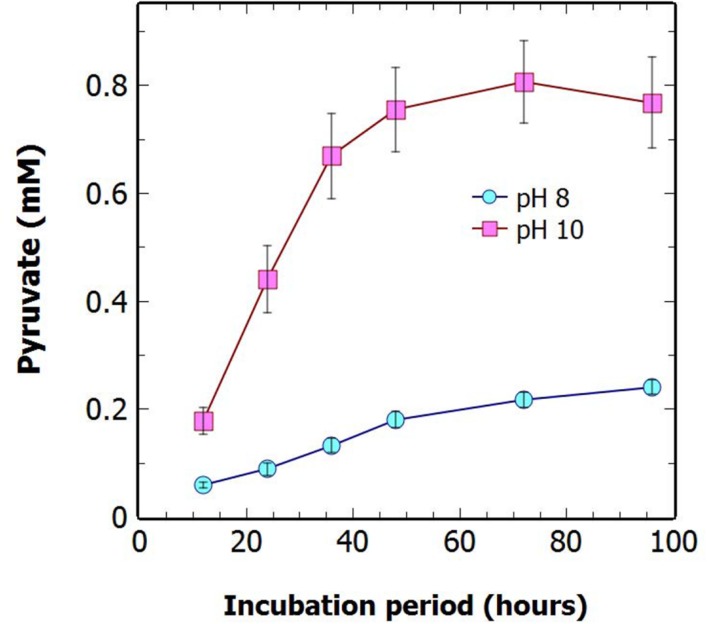
**The effect of pH on extracellular pyruvate levels.** Growth Δ*glgC* under N-deprivation at high pH increases extracellular pyruvate. The extracellular concentration of pyruvate was measured over 96 h of N-deprivation at different media pH. Cultures were standardized to 1 OD_730_ at 0 h N-deprivation for the pH8 and pH10 treatments. Displayed are the averages of technical duplicates of biological quadruplicates. Data points indicate mean ± SE (*n* = 4).

When the pH of the culture media was increased from pH 8 to pH 10 there was a greater than threefold increase in the concentration of pyruvate secreted over 96 h of N-deprivation (**Figure 6**). As seen previously at pH 8, most of the pyruvate was secreted within the first 48 h of N-deprivation at alkaline pH. This enhancement of pyruvate excretion at high pH has not been previously observed in cyanobacteria. There is potential for the existence of an as yet unidentified uptake transporter to be involved in this enhanced efflux at high pH. There was also a tendency for the enhanced pyruvate excretion at high pH to occur at the apparent expense of oxoglutarate levels (**Supplementary Figure S3**).

### Pyruvate Production Increases with Addition of Minimal Amounts of Nitrate across Time Course

The re-addition of low concentrations of nitrate throughout the time course increased pyruvate production and partially boosted cell growth and photosynthetic rate (**Supplementary Figure S4**). N-deprivation was initiated via re-suspension in nitrogen-free BG11_0_ medium. At different time points across the incubation period, pulses of NaNO_3_ were added to a final concentration of 0.05 or 0.1 mM (by comparison, in nutrient sufficient media, the NaNO_3_ concentration in BG11 media was 20 mM). Both pulse concentrations were found to significantly increase the amount of pyruvate secreted compared to the no-nitrate control. The greatest increase (40%) was seen when 0.1 mM pulse concentrations of NaNO_3_ were added at 12 and 24 h (two pulse additions). This was noted in conjunction with a slight stimulation of cell replication and an increase in net oxygen evolution rate 2 h after nitrate addition (results not shown). These results suggest that the addition of low levels of nitrate boosts cell function, whilst still invoking a N-deprivation response, and thus maintaining pyruvate excretion. In future studies it may be possible to balance the degree of N-stress with a more sustained excretion of pyruvate.

## Discussion

### Pyruvate Excretion

The commercial demand for pyruvate continues to expand as it becomes widely used in the drug, agrochemical, chemical and food industries (reviewed by Li et al., 2001). Such pyruvate production is usually derived from *E. coli* bioreactors using glucose as a feedstock, so there is considerable interest in engineering cyanobacterial strains for cheaper production of pyruvate supported by photosynthetic synthesis. Our study describes the redirection of carbon flux in a model cyanobacterial species, *Synechococcus elongatus* PCC 7942, using a glycogen-storage deficient strain (Δ*glgC*) under nitrogen deprivation conditions, to yield the industrially desirable compound, pyruvate. We have shown that this strain is capable of significant pyruvate production, contrary to previous findings (Hickman et al., 2013), and that this excretion can be enhanced by increasing the culture pH, by partial re-addition of nitrogen (N) and by photomixotrophic growth under N deprivation. Although pyruvate excretion has been reported for glycogen deficient strains of *Synechocystis* 6803 under nitrogen deprivation (Carrieri et al., 2012; Grundel et al., 2012) and the euryhaline cyanobacterium, *Synechococcus* sp. Strain PCC7002 (Jackson et al., 2015), this study represents the first time it has been observed in *S. elongatus* 7942. This difference could potentially be related to avoidance of freezing samples, as we found that fast freezing of pyruvate in growth media leads to a significant and variable decrease in measured pyruvate (**Supplementary Figure S2A**). It is possible that, upon freezing, the highly reactive α-keto acids are degraded by trace amounts of oxidizing contaminants not present in pure water (Zimmermann et al., 2014). We discovered that storage of supernatants for as long as 4 days, at 4°C, posed no determination issues (**Supplementary Figure S2C**).

### Source of Excreted Pyruvate

Using BG11_0_ (N-free) medium containing ^13^C-labeled bicarbonate and NMR spectroscopy (Carrieri et al., 2012) it was established that pyruvate and 2-oxoglutarate present in the external medium of *Synechocystis* 6803 Δ*glgC* under N-deprivation, were synthesized directly from newly fixed carbon. However, in our investigation, no positive correlation was identified between the photosynthetic capacity (as a surrogate for net CO_2_ fixation) of Δ*glgC* cells under N-deprivation and the amount of pyruvate produced (**Figure 2**). A similar decline in photosynthetic capacity, under N-deprivation, in a *glgC* mutant of the euryhaline cyanobacterium, *Synechococcus* sp. Strain PCC7002, was recently observed (Jackson et al., 2015). A possible explanation for this lack of correlation is that the supply of reduced carbon could be a limiting factor for pyruvate production and subsequent excretion. To investigate the validity of this possibility, *S. elongatus* Δ*glgC* was engineered to express transporters for glucose and sucrose and grown photomixotrophically with exogenous organic carbon supply. Under conditions of N-depletion and glucose-driven photomixotrophic metabolism, a fourfold increase in the extracellular pyruvate concentration was observed (**Figure 4**). The addition of glucose or sucrose has no effect on pyruvate excretion in the unmodified *glgC* mutant, nor did the expression of the sugar transporters in Δ*glgC* have any measurable effect on pyruvate excretion if the sugars where omitted (results not shown). The apparent lack of entry sucrose or glucose, without the expression of a cognate transporter, was consistent with a study in *Synechococcus* PCC7942 (McEwen et al., 2013) showing that entry of these exogenous compounds was trivial without an expressed foreign sugar transporter. This enhanced rate of pyruvate excretion when Δ*glgC* was engineered to express transporters for glucose or sucrose supports the suggestion that the availability of reduced carbon reserves could be a major limiting factor for pyruvate excretion.

Further investigation revealed a positive correlation between the density of the starting cultures and the pyruvate excretion (**Figure 5**). In late-growth stage cultures, cyanobacterial cells limited by the depletion of nutrients or photon flux, undergo a number of metabolic changes to adapt to these limiting conditions including a decrease in photosynthetic rate and the accumulation of carbon and nitrogen stores (Aguirre von Wobeser et al., 2011). Consequently, late phase cultures have a greater quantity of internal carbon stores than the rapidly replicating, mid-log phase cultures. We observed a correlation between culture density prior to the onset of N-deprivation and subsequent pyruvate excretion (**Figure 5**), which would be at least partially consistent with pyruvate being dependent on reduced carbon reserves.

A recent study in a cyanobacterial strain of *Arthrospira platensis* determined that under N-deprivation that glycogen was not synthesized from carbon derived from CO_2_ (Hasunuma et al., 2013), instead it was deduced that the likely source was carbon derived from the gluconeogenesis of amino acids released from protein degradation. This finding, whilst somewhat supportive our view, causes further complications in that the only significant protein pool in *S. elongatus* 7942 is phycocyanin, which is not broken down under N-deprivation in the non-bleaching Δ*glgC* strain (**Figure 1D**). In fact, a previous study (Hickman et al., 2013) observed a 50% decrease in cellular protein levels in the WT, which was not observed in *glgC* null mutants. Additionally, immediate cell cycle arrest upon nitrogen starvation (**Figure 1A**) suggests an inability to remobilise internal nitrogen stores. It is possible that the carbon for pyruvate synthesis is sourced from the remobilisation of other carbon stores within the cell.

### Optimization of Pyruvate Excretion

Carrieri et al. (2012) suggested that for biotechnological applications product yield could be improved in *Synechocystis* Δ*glgC* by the supply of small quantities of nitrogen during a nitrogen-limited regime. This potential was investigated, and it was found that the addition of small concentrations of nitrate across N-deprivation time course lead to increased pyruvate production in *S. elongatus*-7942-Δ*glgC* (**Supplementary Figure S4**). It is likely that this is due to the associated boost in cellular reallocation of fixed carbon. This investigation of *Synechococcus* PCC7942 indicates there is a potential for further optimization of pyruvate excretion.

A substantial increase in the extracellular concentration of pyruvate was seen when the buffered pH of the media was increased from pH-8 to pH-10 (**Figure 6**). A similar relationship was seen by Ducat et al. (2012) who reported increased sucrose excretion at increasing pH, resulting from a reversal of the direction of sucrose permease (*cscB*), a sucrose/proton symporter at high pH. This suggests the mechanism of pyruvate export from the cell could be due to an unknown substrate/proton symporter, which can be enhanced in an efflux mode by high pH. The mechanism of pyruvate export has not previously been investigated. However, Grundel et al. (2012) established that it was not due to lysis or general leakage effects as no phycobiliproteins or glutamate were detected in the supernatant. This conclusion was supported by the lack of protein in the supernatants in our studies (results not shown). Further investigation is required to determine the specific mechanism of cellular pyruvate export, however, simply increasing the medium pH during N-deprivation in the *glgC* mutant background represents a viable strategy for the further optimizing of pyruvate excretion.

In the future, further optimization of pyruvate excretion is possible once the nature of the carbon source for secreted pyruvate and its mode of transport across the cell membrane are determined. Measurement of the allocation of newly fixed ^13^C-labeled carbon at various time points could be used to quantitatively map intracellular carbon fluxes within the strain. This would help to identify pathway bottlenecks and alternative carbon storage pools (Young et al., 2011), thus indicating targets for metabolic engineering to help optimize the flux toward pyruvate. If the hypothesis that pyruvate is derived from the remobilisation of carbon stores proves correct, it may be worthwhile to return to *Synechocystis* 6803 for further optimization. This strain is known to accumulate cyanophycin protein granules (CPGs) up to 16% of cellular dry weight in stationary phase cells (Allen, 1988). Under N-deprivation CPGs are rapidly degraded (Allen, 1988), and could therefore represent a considerable carbon source for pyruvate synthesis. It might be possible to develop a multi-step system for pyruvate production, where CPG accumulation is triggered via sulfur depletion or the addition of excess-nitrogen containing compounds, before shifting to N-deprivation. The potential of this strategy reveals the importance of establishing the source of carbon so future optimization steps can be targeted at the appropriate metabolic progresses.

## Author Contributions

GP, MD, CH, SB conceived and planned the project. PB, LR, DP-M, TT, GJ carried out experimental work and analyses; primarily PB and DP-M. GP, PB, DP-M, MD, CH, SB wrote, and or commented, on the manuscript; LR, TT, GJ checked the final version.

## Conflict of Interest Statement

The authors declare that the research was conducted in the absence of any commercial or financial relationships that could be construed as a potential conflict of interest.
