# Supplementary material for: Factors Altering Pyruvate Excretion in a Glycogen Storage Mutant of the Cyanobacterium, Synechococcus PCC7942
Source: Front Microbiol. 2016 Apr 5;7:475. doi: 10.3389/fmicb.2016.00475 (PMC4820439; doi:10.3389/fmicb.2016.00475)
Supplement: Supplementary file 1 [file Presentation_1.PDF]

Supplementary Files for: Optimisation of pyruvate excretion in a glycogen storage mutant of the cyanobacterium, *Synechococcus* PCC7942

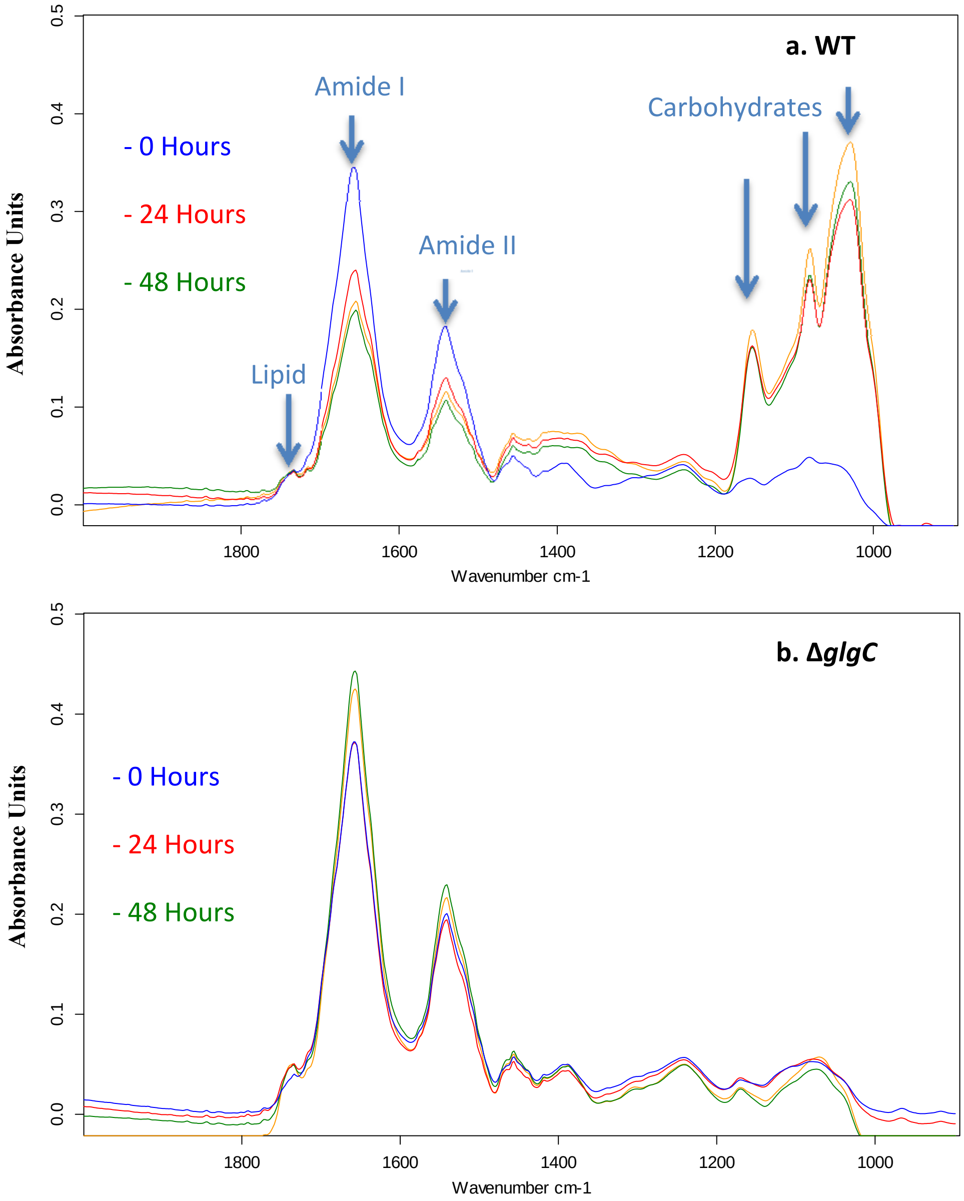

Supplementary Figure 1: FTIR spectrum of *S. elongatus* 7942 (a) WT and (b) Δ*glgC* cells at 0 (blue), 24 (red), 48 (green) and 96 (orange) hours of N-deprivation. Plots represent the average of technical duplicates of biological triplicates. Absorbance was normalized against the lipid absorbance, after baseline correction. Note at 24 and 48 h the mutant is largely devoid of carbohydrate compared to WT cells.

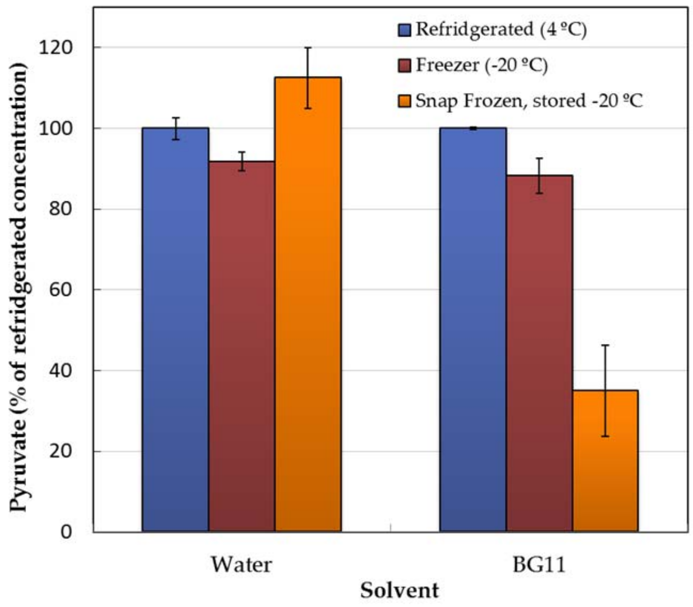

Supplementary Figure 2A: Stability of pyruvate in $BG11_0$ media or water stored at different conditions for 24 hours. Exogenous 10mM sodium pyruvate was added to $BG11_0$ media or distilled water, then aliquots were taken and stored in 1.5mL Eppendorf tubes, at the indicated temperatures. Snap-freezing was achieved by submersion of samples in liquid nitrogen. After 24 hours samples were bought to room temperature and pyruvate concentration was measured. Data indicate mean ± standard deviation (n=3). Note that snap-freezing leads to a variable loss of assayable pyruvate in BG11 media whereas the effect less pronounced by slow freezing at -20°C in BG11 media; the effects were less noticeable in distilled water.

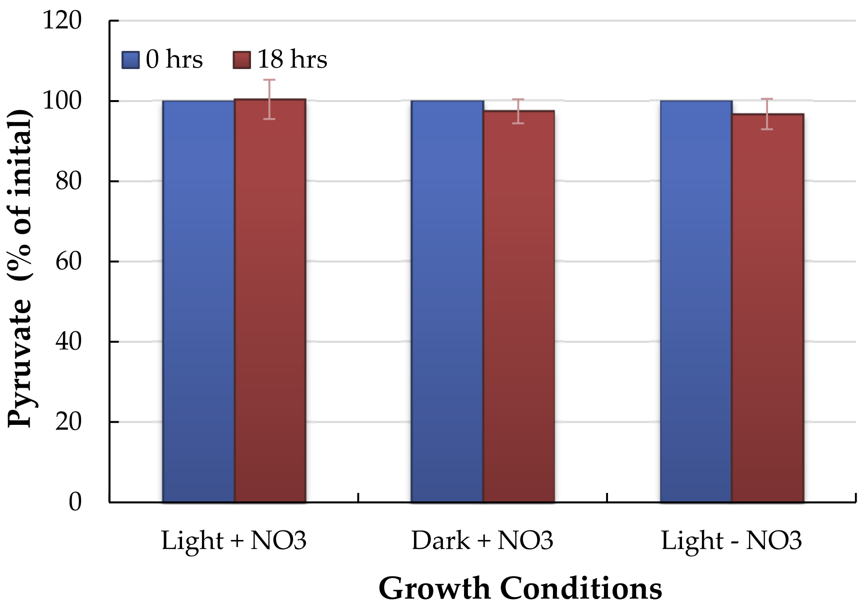

Supplementary Figure 2B: Stability of exogenously-added pyruvate in culture supernatant over 18 hours in different growth conditions with *S. elongatus* 7942 WT cells. At time zero, log phase cells in nutrient sufficient BG11 were centrifuged and resuspended to an OD_{730} of 1.0 ± 0.1 in 100mL BG11/$BG11_0$ media buffered with 20mM HEPES to pH8 and containing 10mM sodium pyruvate. Cultures were aerated with 3% CO_2 at 30°C. Light intensity, where applicable, was 70-75 µE m^{-2} s^{-1}. Pyruvate concentration of supernatant was measured within 1 hour of collection. Data indicate mean ± standard deviation (n=3). Note that exogenous pyruvate is stable for at least 18h in WT cultures in the light or the dark ± nitrate, so external pyruvate is clearly not consumed by the cells.

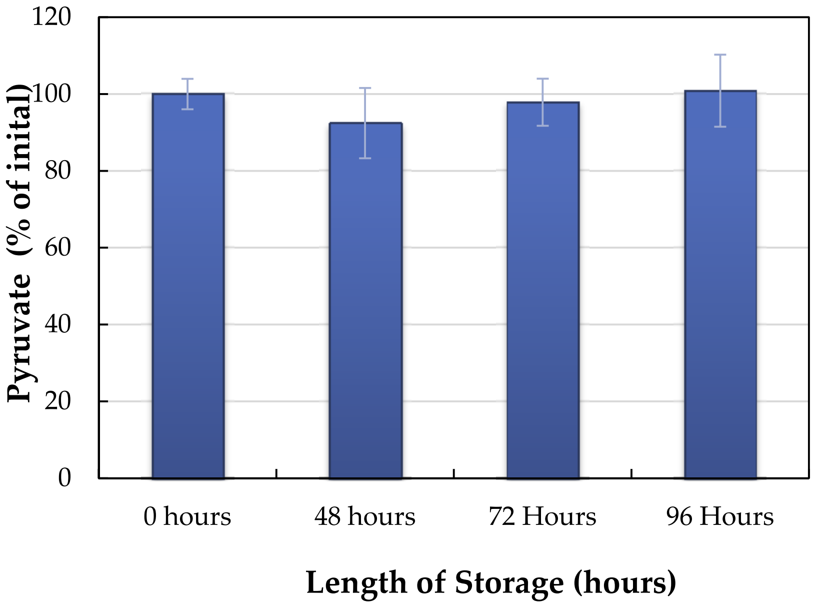

Supplementary Figure 2C: Stability of pyruvate in BG11–NO_3 medium stored at 4ºC at 0, 24, 48 and 96 hours incubation. 10 mM sodium pyruvate was added into BG11 – NO_3 media at time zero. Aliquots were taken and stored at 4ºC in 1.5mL Eppendorf tubes. At 96 hours the pyruvate concentration of the samples was measured with a fluorometric, coupled enzyme assay. Initial pyruvate concentration was 10mM (normalised to 100%). Data indicate mean ± standard deviation (n=3). Note that the pyruvate concentration in BG11 culture supernatant is stable for at least 96 hours storage at fridge temperature.

Supplementary Table 1: FAME content of WT and two mutants, as % DCW, over a 3-day nitrogen deprivation period. Data displayed as means of 3 biological replicates ± SD (n=3).

FAME[a] content as % DCW								
**Day and strain**	**14.0**	**14.1**	**16.0**	**16.1Δ^9**	**17.1**	**18.0**	**18.1Δ^9**	**18.1Δ^{11}**
**Day 0:** **WT**	0.05 ±0.00	0.07 ±0.00	1.45 ±0.00	1.22 ±0.00	0.01 ±0.00	0.05 ±0.00	0.08 ±0.00	0.11 ±0.00
**Δ*glgC***	0.07 ±0.00	0.07 ±0.00	2.93 ±0.08	1.86 ±0.05	0.02 ±0.00	0.54 ±0.02	0.36 ±0.00	0.22 ±0.00
**Δ*glgC*+Δ*sps***	0.04 ±0.02	0.03 ±0.01	1.50 ±0.54	1.11 ±0.41	0.01 ±0.00	0.08 ±0.02	0.17 ±0.06	0.11 ±0.05
**Day 1:** **WT**	0.07 ±0.01	0.08 ±0.02	2.68 ±0.62	1.95 ±0.43	0.03 ±0.00	0.17 ±0.04	0.30 ±0.07	0.34 ±0.07
**Δ*glgC***	0.06 ±0.00	0.06 ±0.00	3.22 ±0.21	1.40 ±0.02	0.03 ±0.00	1.09 ±0.03	0.67 ±0.02	0.23 ±0.01
**Δ*glgC*+Δ*sps***	0.05 ±0.01	0.05 ±0.01	3.62 ±0.67	1.75 ±0.28	0.03 ±0.01	0.98 ±0.18	0.80 ±0.18	0.27 ±0.06
**Day 2:** **WT**	0.04 ±0.00	0.05 ±0.00	1.78 ±0.17	1.36 ±0.13	0.02 ±0.00	0.11 ±0.02	0.23 ±0.03	0.31 ±0.02
**Δ*glgC***	0.05 ±0.00	0.04 ±0.00	3.00 ±0.21	1.16 ±0.02	0.03 ±0.00	1.02 ±0.03	0.69 ±0.02	0.22 ±0.01
**Δ*glgC*+Δ*sps***	0.04 ±0.01	0.05 ±0.01	3.20 ±0.67	1.45 ±0.28	0.04 ±0.01	0.81 ±0.18	0.87 ±0.18	0.28 ±0.06
**Day 3:** **WT**	0.04 ±0.01	0.05 ±0.0	1.48 ±0.22	1.18 ±0.17	0.02 ±0.00	0.10 ±0.02	0.21 ±0.03	0.31 ±0.04
**Δ*glgC***	0.05 ±0.01	0.04 ±0.01	3.40 ±0.38	1.24 ±0.17	0.04 ±0.01	1.21 ±0.18	0.83 ±0.14	0.27 ±0.04
**Δ*glgC*+Δ*sps***	0.02 ±0.02	0.04 ±0.01	3.28 ±0.69	1.47 ±0.39	0.05 ±0.01	0.76 ±0.14	0.91 ±0.20	0.30 ±0.05

[a] The double bond positions in the more abundant FAMEs were determined from the mass spectra of the respective DMOX derivatives.

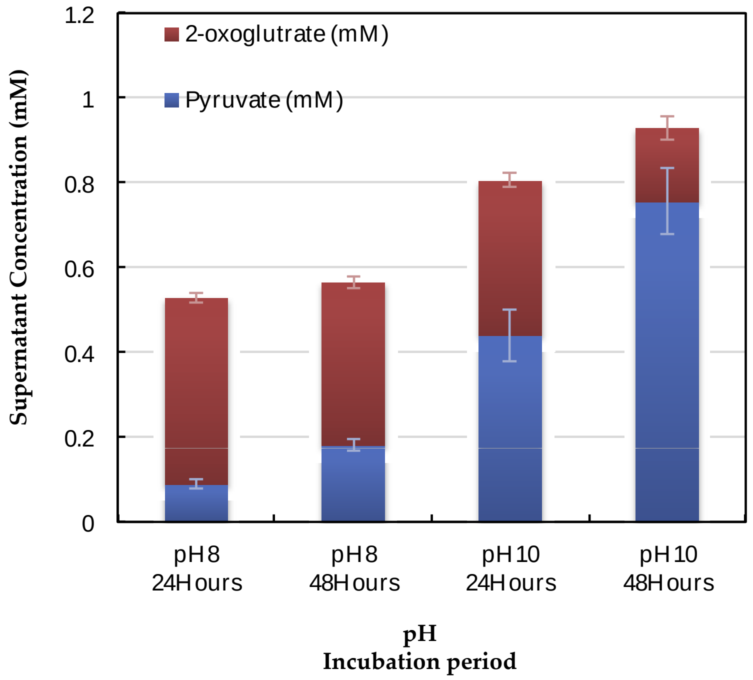

Supplementary Figure 3. Extracellular accumulation of pyruvate and 2-oxoglutarate in *S. elongatus* 7942 *ΔglgC* while N-deprived at either pH 8 or pH 10. Values are normalized to 1 OD_{730} at 0 hours N-deprivation with an OD_{730} range of 0.80-0.90 (pH 8) and 0.87-1.12 (pH 10). Displayed are the averages of technical duplicates of biological quadruplicates. Data indicate mean ± Standard Error (n=4).

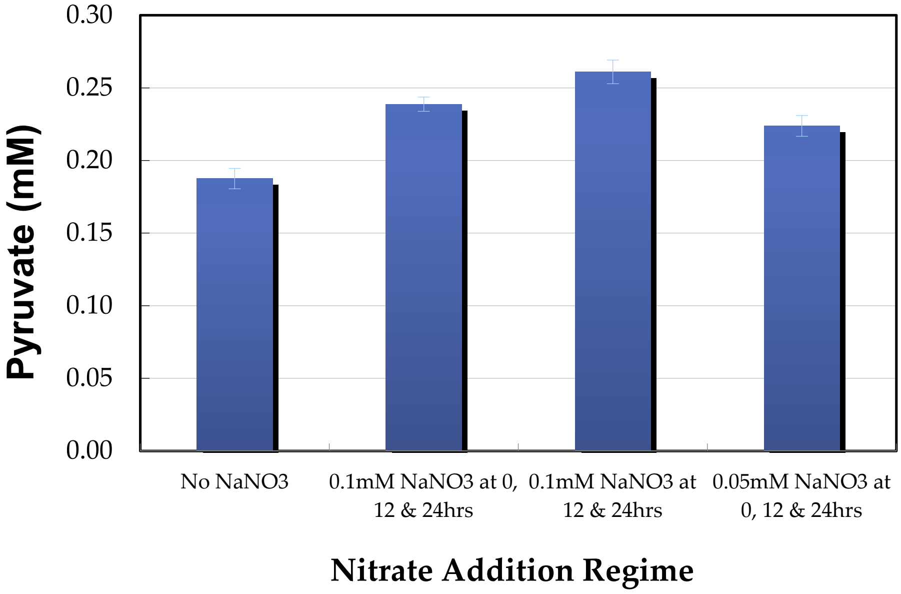

Supplementary Figure 4: Extracellular accumulation of pyruvate in *S. elongatus* 7942 Δ*glgC* in nitrogen-free and nitrogen supplemented media. At 0, 12 and 24 hours, small pulses of $NaNO_3$ (at final levels or 0.05 or 0.1 mM; 50 µL pulse per 100 ml culture) were added to the cultures. Values are normalized to 1 $OD_{730,}$ at 0 hours N-deprivation, with an OD_{730} range of 0.99-1.07. Displayed are the averages of technical duplicates of biological triplicates. Data indicate mean ± Standard Error (n=3).
